# Low-level red laser therapy alters effects of ultraviolet C radiation on *Escherichia coli* cells

**DOI:** 10.1590/1414-431X20154459

**Published:** 2015-07-10

**Authors:** K.S. Canuto, L.P.S. Sergio, O.R. Guimarães, M. Geller, F. Paoli, A.S. Fonseca

**Affiliations:** 1 Centro de Ciências da Saúde, Centro Universitário Serra dos =rgãos, Teresópolis, RJ, Brasil; 2 Departamento de Biofísica e Biometria, Instituto de Biologia Roberto Alcantara Gomes, Rio de Janeiro, RJ, Brasil; 3 Setor de Facomatoses, Serviço de Genética Clínica, Instituto de Puericultura e Pediatria Martagão Gesteira, Universidade Federal do Rio de Janeiro, Rio de Janeiro, RJ, Brasil; 4 Departamento de Ciências Fisiológicas, Instituto Biomédico, Universidade Federal do Estado do Rio de Janeiro, Rio de Janeiro, RJ, Brasil; 5 Departamento de Morfologia, Instituto de Ciências Biológicas, Universidade Federal de Juiz de Fora, Juiz de Fora, MG, Brasil

**Keywords:** DNA, *Escherichia coli*, Laser, Ultraviolet radiation

## Abstract

Low-level lasers are used at low power densities and doses according to clinical protocols supplied with laser devices or based on professional practice. Although use of these lasers is increasing in many countries, the molecular mechanisms involved in effects of low-level lasers, mainly on DNA, are controversial. In this study, we evaluated the effects of low-level red lasers on survival, filamentation, and morphology of *Escherichia coli*cells that were exposed to ultraviolet C (UVC) radiation. Exponential and stationary wild-type and *uvrA*-deficient*E. coli* cells were exposed to a low-level red laser and in sequence to UVC radiation. Bacterial survival was evaluated to determine the laser protection factor (ratio between the number of viable cells after exposure to the red laser and UVC and the number of viable cells after exposure to UVC). Bacterial filaments were counted to obtain the percentage of filamentation. Area-perimeter ratios were calculated for evaluation of cellular morphology. Experiments were carried out in duplicate and the results are reported as the means of three independent assays. Pre-exposure to a red laser protected wild-type and *uvrA*-deficient *E. coli* cells against the lethal effect of UVC radiation, and increased the percentage of filamentation and the area-perimeter ratio, depending on UVC fluence and physiological conditions in the cells. Therapeutic, low-level red laser radiation can induce DNA lesions at a sub-lethal level. Consequences to cells and tissues should be considered when clinical protocols based on this laser are carried out.

## Introduction

Laser devices are monochromatic, collimated, and coherent radiation sources. These devices have been used with different purposes for treatment of many diseases in soft and bone tissues at varying power densities, doses, and wavelengths (1). Low-level laser therapies are used at low power densities and doses, in the so-called therapeutic window (600-1100 nm), in pre-established clinical protocols supplied with laser devices or based on professional practice.

Although use of low-level laser therapy is increasing in many countries, there are questions regarding the molecular mechanisms involved in the effects of this therapy. Molecular targets (chromophores) appear to be some mitochondrial cytochromes and porphyrins in the cytoplasm (2). Laser radiation energy is absorbed by chromophores and subsequent intracellular transducers are responsible for transforming the laser radiation energy into a cellular signal (3). A cascade of molecular effects occurs as a consequence of amplification of the photosignal, including an increase in nucleic acids (4) and ATP (5), as well as gene transcription (6). These alterations increase metabolism, protein secretion, and cellular division after low-level laser exposure (5). The entire effect (biostimulation or biomodulation) is considered the basis of therapeutic applications, such as wound healing (7). For other applications, such as pain relief and herpes simplex treatment, the molecular mechanisms are not understood.

Few studies have evaluated the effects of low-level laser radiation on DNA and the possible consequences to cells and tissues, and whether this radiation induces molecular damage. In fact, low-level lasers at therapeutic doses can induce free radical generation (8) and sub-lethal DNA lesions (9). Additionally, these lasers induce different SOS responses in cells deficient in DNA repair mechanisms (2,10).

Ultraviolet radiation absorption by DNA molecules results in pyrimidine dimers as the main direct DNA lesion, while absorption by other molecules causes free radical generation. These chemical species induce different types of lesions in DNA, mainly with nitrogen bases, by oxidizing chemical reactions (11). Pyrimidine dimers and oxidizing DNA lesions induced by ultraviolet radiation are repaired by the nucleotide excision repair pathway (11). Cells that are deficient in nucleotide excision repair fail to remove pyrimidine dimers and other bulky lesions caused by ultraviolet radiation exposure, similar to humans presenting with xeroderma pigmentosum. *Escherichia coli* has three proteins (uvrA, uvrB, and uvrC) involved in recognizing the lesion and incision endonuclease function (11). *E. coli* cells that are deficient in these proteins are used as experimental models to evaluate cellular responses to ultraviolet radiation (11). However, previous studies have shown that cells exposed to increased free radical concentrations are more resistant to ultraviolet radiation (12). Moreover, previous results in our laboratory have shown that a low-level red laser induces resistance to hydrogen peroxide (9) and induces filamentation in *E. coli* cells deficient in repair of oxidative DNA lesions (10).

Therefore, the effects of low-level lasers on DNA molecules by oxidative mechanisms are still controversial. This study evaluated the effects of a low-level red laser on survival, filamentation, and morphology of *E. coli* cells that were deficient in nucleotide excision repair and were exposed to ultraviolet C (UVC) radiation.

## Material and Methods

### Low-level red laser and UVC source

A therapeutic, low-level red laser (AlGaInP, 10 mW), with emission at 658 nm, was purchased from HTM Eletrônica (Brazil). UVC radiation was produced from a germicidal lamp (Philips, The Netherlands). Table 1 shows parameters of the laser.

**Table:**
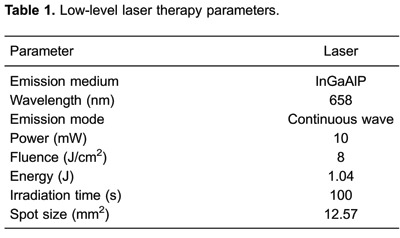


### Evaluation of low-level red laser exposure on survival of *E. coli* cells with UVC radiation

Cultures of *E. coli* AB1157 (wild-type) and AB1886 (*uvrA*-deficient) in the stationary and exponential growth phases were exposed to a low-level red laser (8 J/cm^2^) and UVC radiation (25, 50, and 100 mJ/cm^2^). The rate of survival was evaluated. The laser device controlled laser fluence and irradiation time. UVC fluence was measured by an ultraviolet radiometer (Instrutherm, Brazil) and irradiation times were 25, 50, and 100 s. The laser device was positioned so that the laser beam covered almost all of the surface of bacterial suspension aliquots. Aliquots of bacteria from frozen stocks were used and further incubated in nutritive medium to reach exponential growth (10^8^cells/mL, 2-3 h, 37°C). Other experiments were carried out with cultures of the same *E. coli* strains in the stationary growth phase (10^10^ cells/mL, 18 h, 37°C). Bacterial cells were centrifuged (700 *g*, 15 min) and suspended twice in saline (0.9% NaCl). Aliquots (50 µL, n=5, for each fluence) of bacterial suspensions were then exposed to the low-level red laser and UVC. Bacterial suspensions that were not exposed to the laser or UVC were used as controls. Bacterial suspensions were spread onto Petri dishes. Colonies that formed after overnight incubation at 37°C were counted. The survival fraction was then calculated, and the laser protection factor was calculated by the ratio between the number of viable cells after exposure to the red laser and UVC and the number of viable cells after exposure to UVC.

### Bacterial filamentation assay

To evaluate induction of filamentation, exponential and stationary *E. coli* AB1157 and AB1886 cultures were obtained and exposed to a low-level red laser and UVC as described above. Bacterial suspensions that were not exposed to a laser or ultraviolet radiation were used as controls. Immediately after exposure, aliquots (20 µL) were withdrawn, spread onto microscopic slides, and stained by the Gram method (13). Bacterial cells (100 cells per field, three fields per slide, two slides per group) were visualized by a Carl Zeiss microscope (Germany) equipped with an A-plan 40× objective, a 0.90 condenser, and a 100-W halogen lamp. The images were captured with an AxioCam HRc Sony 12M color microscopy camera, using Axiovision software (Carl Zeiss). The images were then analyzed by Image Proplus software (version 6.0 for Windows XP, Microsoft Corporation, USA) to determine the percentage of bacterial filamentation. A bacterial filament was considered as 2.5 times the average area of the bacterial cells. Experiments were carried out in duplicate and the results are reported as the means of three independent assays.

### Statistical analysis

Data are reported as means±SD of the protection factor, percentage of filamentation, and area-perimeter ratio. One-way analysis of variance followed by Tukey’s *post hoc* test were performed to determine statistical differences, with P<0.05 as the least significant level.

## Results

### Survival of *E. coli* cells exposed to low-level red laser and UVC radiation

Table 2 shows the protection factors for low-level red laser radiation on *E. coli* AB1157 cells, which were exposed to different UVC radiation levels, in the exponential growth phase. There was no significant (P&0.05) protection of the low-level red laser on*E. coli* AB1157 cultures against the lethal effect of UVC radiation.

**Table:**
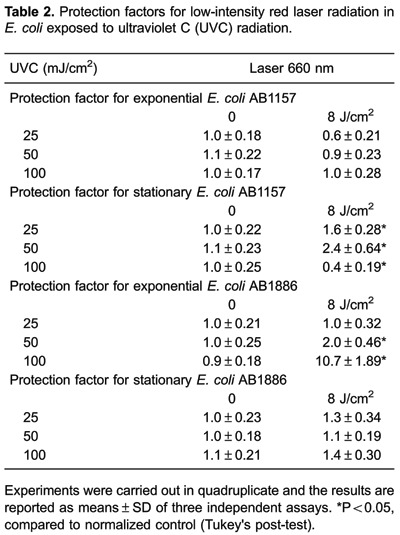


To determine whether the growth phase interferes with laser-induced biological effects, *E. coli* cultures in the stationary growth phase were exposed to UVC radiation after red laser exposure. Table 2 shows the protection factors of the red laser on*E. coli* AB1157 cultures that were exposed to UVC in the stationary growth phase under the same conditions used to irradiate cultures of this strain in the exponential growth phase. In contrast to the exponential growth phase, low-level red laser radiation significantly protected stationary*E. coli* AB1157 cultures against the lethal effect of UVC radiation at lowest fluence levels (P<0.05 *vs*controls). However, at the highest fluence level (100 J/cm^2^), exposure to the red laser decreased the protection factor significantly (P<0.05), compared with *E. coli* cultures exposed to UVC (controls).

Pre-exposure to low-level red laser radiation was evaluated in *E. coli* AB1886 cultures that were exposed to UVC radiation (Table 2). In contrast to wild-type*E. coli* (AB1157), laser pre-exposure (8 J/cm^2^) induced significant (P<0.05) protection against the lethal effect of UVC radiation on *E. coli* AB1886 at the highest fluences used (50 and 100 mJ/cm^2^).

Laser-induced protection of the lethal effect of UVC was evaluated in stationary*E. coli* AB1886 cultures (Table 2). In this condition, the red laser did not significantly protect *E. coli* AB1886 cells against the lethal effect of UVC radiation (P&0.05).

### Induction of filamentation in *E. coli* cells exposed to low-level red laser and UVC radiation

Induction of filamentation was evaluated in exponential *E. coli*AB1157 cultures pre-exposed to low-level red laser radiation and exposed to UVC radiation (Table 3). Exposure to UVC radiation significantly increased the percentage of bacterial filamentation (P<0.05), compared with *E. coli* cultures exposed to UVC (controls). Similar percentages of bacterial filamentation were observed in cultures pre-exposed to the red laser (8 J/cm^2^). Percentages of bacterial filamentation were not significantly different from those in*E. coli* AB1157 cultures exposed to laser alone (P&0.05), except in cultures exposed to UVC radiation at the lowest fluence (25 mJ/cm^2^).

**Table:**
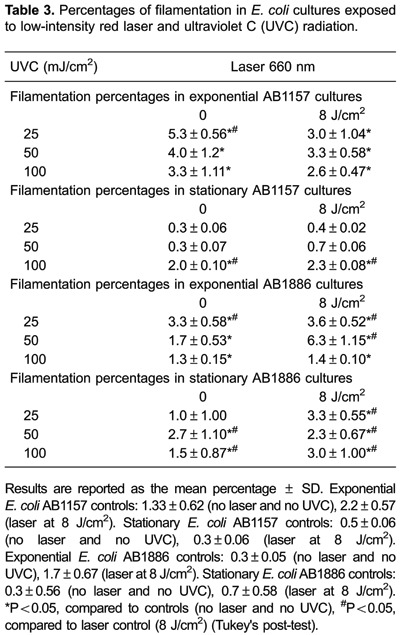


The percentage of filamentation in the stationary growth phase of *E. coli* AB1157 cultures did not significantly change after UVC exposure at the lowest fluences (25 and 50 mJ/cm^2^) (P&0.05, Table 3). However, the percentage of bacterial filamentation was significantly higher at the highest fluence (100 mJ/cm^2^) compared with controls (no laser and no UVC) and compared with the laser alone (P<0.05).

The percentage of bacterial filamentation in exponential *E. coli*AB1886 cultures that were exposed to UVC radiation is shown inTable 3. Exposure to UVC radiation significantly induced filamentation in non-pre-exposed and pre-exposed low-level red laser radiation (P<0.05). These percentages of bacterial filamentation were similar to those induced by the red laser alone (P&0.05).

Except for the lowest UVC fluence (25 mJ/cm^2^), non-laser pre-exposed and laser pre-exposed stationary *E. coli* AB1886 cultures had significantly higher percentages of bacterial filamentation (P<0.05), compared with *E. coli* cultures exposed to UVC (controls). These percentages of bacterial filamentation were significantly higher than those observed in stationary *E. coli* AB1886 cultures that were exposed to the red laser alone, except for UVC alone at the lowest fluence (P<0.05, Table 3).

### Morphology of *E. coli* cells exposed to low-level red laser and UVC radiation

**Figure 1:**
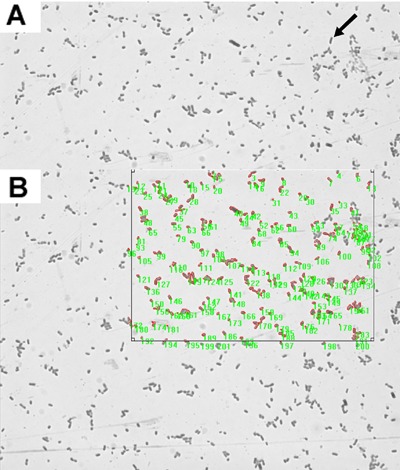
Representative image of bacterial filamentation in an AB1157 culture in the exponential growth phase. *A*, The arrow indicates bacterial filamentation; *B*, inset shows how image analysis was performed. A bacterial filament was considered present when the area of a bacterial cell was larger than 2.5 times the mean area of bacterial cells.

Figure 1 shows representative cells from*E. coli* AB1157 cultures in the exponential growth phase (1A) and analysis of bacterial cells (1B). The area-perimeter ratio of*E. coli* AB1157 cells in the exponential growth phase, exposed to UVC after exposure to low-level red laser radiation, was not significantly (P&0.05) altered (data not shown). Similarly, red laser and UVC radiation alone, or red laser followed by UVC radiation, did not significantly (P&0.05) alter the area-perimeter ratio of *E. coli* cells in the stationary growth phase.

**Figure 2:**
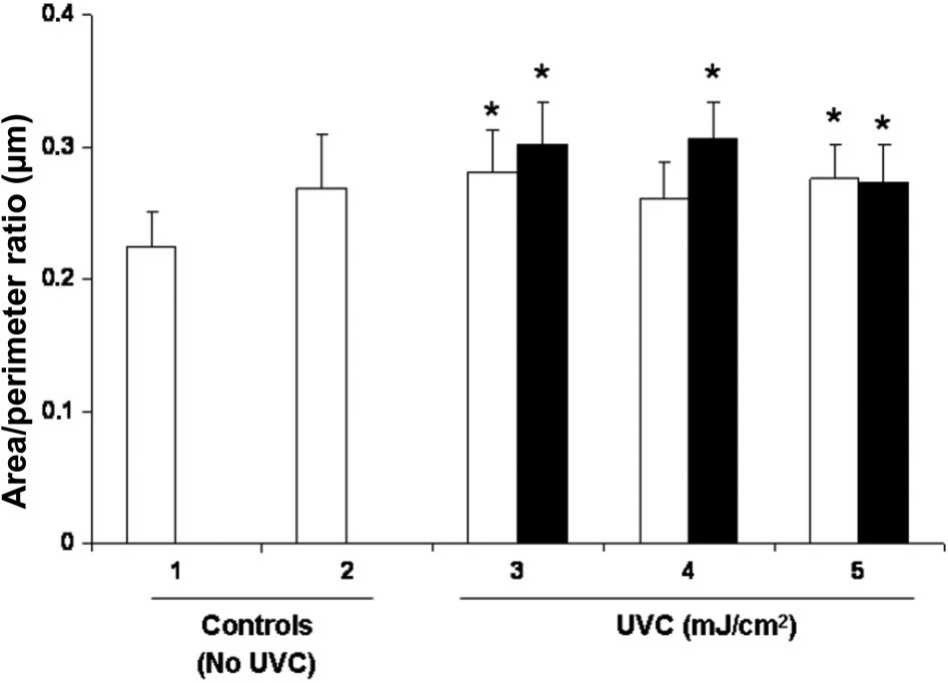
Area-perimeter ratio of exponential *Escherichia coli*AB1886 cells pre-exposed to a low intensity red laser and UVC radiation.*1*) Non-irradiated control,*2*) red laser at 8 J/cm^2^,*3*) UVC at 25 mJ/cm^2^ (white bar) and red laser at 8J/cm^2^+UVC at 25 mJ/cm^2^ (black bar),*4*) UVC at 50 mJ/cm^2^ (white bar) and red laser at 8 J/cm^2^+UVC at 50 mJ/cm^2^ (black bar), and *5*) UVC at 100 mJ/cm^2^ (white bar) and red laser at 8 J/cm^2^+UVC at 100 mJ/cm^2^(black bar). Experiments were performed in duplicate and the results are reported as means±SD of three independent assays. *P<0.05, compared to control 1 (non-irradiated cells) (one-way analysis of variance, followed by Tukey’s *post hoc*test).

Effects of low-level red laser and UVC radiation on the area-perimeter ratio of exponential *E. coli* AB1886 cells were also examined (Figure 2). Exposure to UVC radiation after pre-exposure to the red laser significantly increased the area-perimeter ratio, at least at the lowest UVC fluences (25 and 50 mJ/cm^2^, P<0.05). However, this effect was not observed in stationary *E. coli*AB1886 cells because the area-perimeter ratio was not significantly (P&0.05) altered (data not shown).

## Discussion

Our study showed that low-level red laser radiation, at the fluence used for therapeutic applications, did not protect exponential wild-type *E. coli* cells (AB1157) against the lethal action of UVC radiation (Table 2). In stationary wild-type *E. coli* cells, the effect of the red laser was dependent on UVC fluence, presenting a protective effect at the lowest UVC fluences and a synergistic effect with UVC radiation (Table 2). In exponential*uvrA*-deficient *E. coli* cells (AB1886), the red laser induced protection against the lethal effect of UVC radiation (Table 2). However, laser-induced protection in*uvrA*-deficient cells was larger than that observed in wild-type*E. coli* cells. This result is in accordance with previous observations that exposure to low-level red lasers induces sub-lethal lesions in DNA molecules (9). The He-Ne laser (632.8 nm) protects wild-type and *uvrA*-deficient *E. coli*cells against UVC radiation (2,14). In our study, pre-exposure to low-level red laser radiation increased the lethal effect of UVC radiation*E. coli* in the stationary growth phase. This finding reinforces that laser-induced effects might be different when physiological conditions in cells are modified. In addition, laser radiation parameters, such as wavelength, fluence, and irradiance, can determine the biological effects. Laser-induced protection could be dependent on UVC fluence and physiological conditions in cells, because in our study, pre-exposure to a red laser did not alter the survival of an exponential wild-type *E. coli* strain (AB1157) at all UVC fluences evaluated. In fact, a previous study showed that low-level laser effects depend on physiological conditions in the cells (15). However, in our study, laser protection against the lethal effect of UVC radiation was not observed in stationary *uvrA*-deficient *E. coli* cells (Table 2). This result is in agreement with previous studies showing that laser-induced effects depend on physiological conditions in cells ([Bibr B16] [Bibr B17] 1618).

To determine whether laser-induced protection against effects of UVC radiation involve other DNA repair mechanisms, we evaluated induction of filamentation. Exposure to UVC radiation induced similar percentages of filamentation in wild-type*E. coli* cultures that were not exposed and pre-exposed to a low-level red laser, but the percentage of filamentation was larger in exponential cultures than in stationary cultures in both non- and pre-exposed to a red laser with UVC (Table 3). However, in*uvrA*-deficient *E. coli* cultures, in the exponential and stationary growth phases, pre-exposure to a low-level red laser increased the percentages of filamentation at some UVC fluences (Table 3). Filamentation is part of the SOS response, which is a set of physiological and biochemical modifications in response to DNA damage induced by chemical and physical agents (11). There are a few studies on induction of the SOS response in prokaryotic cells exposed to low-level lasers (10,15,16,18). However, no studies have shown induction of an SOS response by low-level lasers followed by UVC radiation. Laser-induced SOS responses in *E. coli* cultures have been observed by induction of *phr* gene expression (2,19). Previous studies have shown that low-level red and near-infrared lasers induce filamentation in exponential and stationary *E. coli* cultures (10,16,18). Therefore, our finding of filamentation in cells that were pre-exposed to a laser is in agreement with those previous studies. The highest percentage of filamentation observed in*uvrA*-deficient *E. coli* cells could be explained by a possible synergistic effect of the low-level red laser and UVC radiation. In addition, a larger induction of filamentation in *E. coli* cultures pre-exposed to a red laser could explain the highest survival of these cells exposed to UVC radiation.

The filamentation phenotype can be induced in part among stressed cells in a prokaryotic culture in response to an aggressive agent (20). However, quantification of bacterial filaments does not take into account non-filamentous cells. To evaluate this in our study, the area and perimeter of cells were measured after low-level red laser and UVC radiation exposure. We found that the laser alone or laser use prior to UVC radiation exposure did not alter the area-perimeter ratio of exponential and stationary wild-type*E. coli,* suggesting no morphological alteration of cells. However, *uvrA*-deficient *E. coli* cells in the exponential growth phase had an increased area-perimeter ratio after red laser pre-exposure and UVC exposure (Figure 2). This result could be related to a protective effect of the red laser against the lethal effect of UVC radiation and the higher percentages of filamentation obtained in exponential *uvrA*-deficient *E. coli* cells (Tables 2 and 3). Additionally, results from area/perimeter ratio of non- and pre-exposed to a red laser with UVC (Figure 2) are in concordance with the hypothesis that biological low-level laser effects are dependent on DNA repair mechanisms (21). Similar to wild-type *E. coli*, the area-perimeter ratio in stationary *uvrA*-deficient *E. coli* cultures was not altered. This finding indicates that laser-induced effects on UVC action are dependent on the physiological condition in cells, at least among cells that are deficient in DNA repair mechanisms.

Taken together, our results suggest that low-level red laser exposure induced free radical generation in biological systems, which induced protective mechanisms against UVC radiation. This could be part, or a consequence, of a laser-induced biostimulation effect, leading to higher cell survival and regeneration in damaged tissues submitted to low-level laser therapy.

Our results showed that a low-level red laser protected cells against the lethal effect of UVC radiation, and induced filamentation and morphological alterations, depending on DNA repair mechanisms and physiological conditions in cells. Therapeutic low-level red laser radiation can induce DNA lesions at a sub-lethal level. Consequences to cells and tissues should be considered when clinical protocols based on this laser are carried out.
